# Estimation of Post-Cracking Dissipation Capabilities of Fiber Reinforced Concretes in Three Point Bending Test Monitored with Application of Digital Image Correlation System

**DOI:** 10.3390/ma14175088

**Published:** 2021-09-06

**Authors:** Duyen Trinh-Duc, Andrzej Piotrowski, Cezary Ajdukiewicz, Piotr Woyciechowski, Marcin Gajewski

**Affiliations:** Institute of Building Engineering, Warsaw University of Technology, Armii Ludowej 16, 00-637 Warsaw, Poland; duyen.trinh_duc.dokt@pw.edu.pl (D.T.-D.); apio@il.pw.edu.pl (A.P.); c.ajdukiewicz@il.pw.edu.pl (C.A.); p.woyciechowski@il.pw.edu.pl (P.W.)

**Keywords:** fiber reinforced concrete, 3PB test, DIC, ARAMIS, post-cracking dissipation

## Abstract

Concretes with dispersed reinforcement are increasingly used in structural engineering. The basic source of knowledge on their application and design are the Model-Code 2010 guidelines. These guidelines, however, apply mainly to steel rebar reinforcement and are not fully sufficient in the analysis of the load-bearing capacity of elements made of concrete with dispersed reinforcement. Therefore, scientific research in this field is carried out continuously. The main goal of our work is to provide experimental data for the calibration of constitutive models of the cracking mechanics of concrete with reinforcement in the form of steel and polypropylene fibers. This article shows the possibility of using the digital image correlation system (DIC) to achieve this goal. The method of sample preparation and the method of conducting the tests were modeled on the recommendations contained in the PN-EN 14651: 2007 standard. The tests were carried out on prismatic elements with a notch loaded in a three-point bending setup. The results of standard strength tests are presented in the form of column graphs and tables. As an extension, the results of calculations of energy dissipated in fracture process are given. Moreover, the experimentally obtained graphs of the relationship between the force, displacement and crack opening were presented, which were supplemented with the images of crack development obtained with the use of DIC. The development of the crack net is characterized not only qualitatively but also quantitatively as a function of deflection or crack mouth opening displacement. Conclusions concerning the adopted research methodology and the tested materials are presented at the end of the article.

## 1. Introduction

Concrete reinforced with various types of fibers is very suitable materials for many specific construction applications. Fiber-reinforced concretes are composite materials, the properties of which depend not only on the characteristics of the constituent elements, i.e., the concrete including aggregate, binder and the fibers used but also on their arrangement in the volume of the material, and the nature of the interaction between the concrete mixture and dispersed reinforcement [1]. Of course, concrete itself can also be seen as a composite material, but for the purposes of this article, we will focus only on the material that we understand as a concrete non-homogenous matrix and the fibers dispersed in it that constitute the reinforcement. For steel bar reinforced concrete, the design methods are highly developed and generally accepted. It is usually assumed that the load-bearing capacity of the elements reinforced with bars is determined by the positive physical-mechanical features of the constituent materials, i.e., the compressive strength of concrete and the tensile strength of steel. The methods of designing concrete structures with dispersed reinforcement are not so well established and it is necessary to constantly improve them [2]. There are no formal regulations in Eurocodes, and the basic source of knowledge in this regard is Model Code 2010 (fib 2010) [3], which covers the issues related to dispersed steel reinforcement.

According to this document, the basis for designing is knowledge about the residual properties of the material after cracking. The classification of fiber reinforced concrete (FRC) in this respect concerns mainly the residual flexural tensile strength Fr1 and Fr3 (which is defined precisely later), while the numeral symbol index refers to the width of the *CMOD* (crack mouth opening deflection) scratch (1 and 3 mm, respectively) in the bending test of a freely supported beam with a cut in its axis of symmetry (according to EN 14651 standard). The guidelines from the Model Code 2010, however, are not fully sufficient to design a structure made of FRC ensuring the effective use of the properties of this material.

Currently, the most rational method of designing FRC structures is to apply the finite element method [4,5,6,7,8]. Therefore, it is necessary to develop constitutive models of materials reinforced with dispersed fibers and rationally determine the parameters for these models [5,9,10,11,12,13]. In this article, we are focused on the presentation of the results of research carried out for FRC with steel and polypropylene fibers.

The addition of fibers to concrete significantly affects most of its properties, including both the properties of the concrete mix and hardened concrete. Generally, the relationships between the composition of plain concrete and its properties, such as the influence of *w*/*c* ratio and cement content on strength, shrinkage and durability characteristics, are confirmed for FRC also. However, the presence of fibers modifies these relationships, primarily due to a significant deterioration of workability with increasing fiber content, regardless of the fiber material (steel, synthetic) [14,15]. The deterioration of the workability is also associated with the increase in the length of a single fiber. As a result, the porosity of the concrete mix tested with the pressure method increases with the increase in the proportion of fibers in the mix, which is a result of deterioration of workability and difficulties in effective mix compaction [16,17,18,19]. This effect is also visible during the analysis of the microstructure of the hardened concrete—an increase in the macro-porosity of the hardened concrete is observed and the pores are located in the vicinity of larger clusters of fibers [18]. The effect of additional macropores in FRC is particularly pronounced in relation to concretes with a high fiber content [20], in which the compressive strength decreases compared to plain concrete. At low fiber contents, workability disturbances are minor and do not lead to this effect. In test results of other mechanical properties, such as tensile strength, bending, modulus of elasticity, energy of destruction—an increase in these properties is observed along with an increase in the fiber content, unless their critical content is exceeded due to workability, which prevents correct compaction [15]. The critical fiber content is defined in many publications and depends primarily on the slenderness of the fibers (l/d) and the maximum aggregate grain (the smaller the maximum grain, the higher the critical fiber content) [21,22,23,24].

Fibers added in concrete mainly provide crack control due to the capability of crack-bridging after cracking, which means tensile stress transfer across crack. In effect, fibers provide significant resistance to shear across developing cracks. This effect is strong for steel fibers, because SFRC demonstrates a pseudo-ductile behavior, increased residual strength and enhanced energy dissipations capacities, in comparison with the brittle behavior of plain concrete. In that light, fibers have been proved as a promising non-conventional reinforcement in concrete elements under shear stresses due to the beneficial cracking performance of SFRC and, under specific circumstances, could alter the brittle shear failures to ductile flexural one. According to [24] fiber-reinforced concrete (FRC) can be subdivided into two categories based on the post-cracking behavior: the ordinary FRC which shows a softening behavior after the first peak load where a single major crack formed within the FRC and high-performance FRC (HPFRC), which shows strain-hardening behavior with multiple cracks as the tensile stress attained by fibers is higher than the cracking strength of the concrete matrix. To evaluate the post-critical behavior of SFRC in tension many tension models were proposed [25,26,27,28,29]. The effect depends on characteristics and content of fibers and in [30] found that fiber factor is a more efficient parameter than the volume fraction for the evaluation of the steel fiber contribution. Fiber factor is defined as the result of multiplying three values: fiber volume in matrix, slenderness ratio of the fiber and fiber bond factor, which depends on the shape of the fiber.

In the beginning of presented research, standard test results for FRC (i.e., compressive strength, tensile strength at splitting, initial elasticity modulus) are shown. The scope of experiment includes one dosage of polypropylene fibers and two dosages of steel fibers but all the dosages are in the acceptable range from the point of view of critical workability. The main goal is to present the results of the three-point bending test of beam elements with cutting, used to determine the LOP (limit of proportionality) and residual tensile bending strength [27,31]. The interpretation of these results on the basis of continuum mechanics leads to the conclusion that after the crack initiation, it is possible to trace the energy dissipation accompanying the crack growth [32,33]. The result of such a test can be an excellent material for the verification of constitutive models of fiber-reinforced concretes, which are formulated within the framework of the fracture mechanics [34,35]. An additional element that makes this approach unique is the use of measurement methods based on the optical image correlation (DIC ARAMIS measurement system) to track the development of a crack [36,37]. Thanks to this, it is possible not only to track the force as a function of displacement (or crack opening) but also as a function of the current length of the crack (crack net). Similar experimental methods were used in paper [38], where DIC was applied to analyze the influence of strengthening systems on bond behavior and ultimate capacity of notched concrete beams under different environmental conditions. In the abovementioned paper, the observational range was limited to the region where strengthening fiber-reinforced polymer strips were placed. In the case of our paper and the cited article, extending the research to microstructural observations accompanying cracking would expand the possibility of inferring the course of this process in relation to the properties of the fiber-concrete interface. In the future, we plan to carry out such tests, but here we limit ourselves to determining mainly the mechanical properties of the material without reference to its microstructure.

The main goal of this article is to prepare an experimental material that allows for the validation/verification of constitutive models of fiber reinforced concrete in the so-called post-failure phase of their work. From the point of view of fracture mechanics, establishing the relationship between the amount of dissipated energy and the length of the crack is a fundamental issue.

## 2. Materials for Testing and Evaluation

In this experiment, four types of fiber-reinforced concrete samples were used to investigate and evaluate post-cracking dissipation in a three-point bending test compared with the control samples of concrete without reinforcement. The materials used in matrix composite basically were the same while the type and the percentage of the volume of fibers were varied.

Two different types of fibers were used: Poly Propylene (PP) and Steel fiber Dramix 4D-80/60BG. The main geometrical and mechanical properties of the two fibers are shown in Table 1. The two types of fibers were mixed with concrete at different weight ratios per cubic meter: 1.64 kg/m^3^ for PP, and 25 kg/m^3^, 75 kg/m^3^ for steel fibers. Portland cement CEM I 42,5R was used as a binder. Vistula River sand (0/2 mm) and granite as a coarse aggregate (2/8 and 8/16 mm) were also used.

The density and properties of fibers used in the experiment for cementitious composite in the total volume of 1 m^3^ is presented in Table 1. Table 2 presents the composition of concrete mixes with their symbols (M0 to M4) and the volume share of the fibers.

The order of compounds dosing and mixing procedure was as follows:

- Coarse and fine aggregate dosing;
- Short mixing;
- Cement dosing;
- Two minutes of mixing in dry state;
- Dosing of water and superplasticizer;
- Mixing with water and dosing fibers up to 5 min.

Molding of specimens was done according to the EN 12390-1 standard. After 24 h of curing specimens covered with plastic sheet at temperature equal to 20 °C, they were demolded and cured in water till the testing date (up to 56 days).

## 3. Experimental Procedure

### 3.1. Standard Tests

As standard tests of the analyzed fiber-reinforced concretes, the compressive strength was determined on cubic samples with side of 150 mm in accordance with EN-12390-1, EN-12390-3 standards. The splitting tensile strength was also determined on samples of the same dimensions in accordance with EN-12390-6. According to the annex of European Standard EN 12390-6 the cubic specimens are acceptable for splitting tests; however, the values obtained for cubes are approximately 10% higher than for cylinders. The Authors’ laboratory experience shows that tests on 150/300 mm cylinders and 150 mm cubes made of plain concrete leads to the very close results. For this reason, cubic specimens were used for tests of both plain and SFR concretes. The aim of the test was to compare the values for both types of tested concrete and in our opinion the relation between SFRC and plain concrete would be the same when compare results for cylinders. Moreover, the modulus of elasticity was determined on the cylindrical samples in accordance with the EN-12390-13. These tests were not the main goal of this study, and the compilation of their results is intended to present the general mechanical properties of the analyzed materials. Some more detailed analysis on that regard may be found for example in [39]. Additionally, thanks to these data, the results presented in this article can be used for the calibration of constitutive models of fiber reinforced concrete.

**Figure 1:**
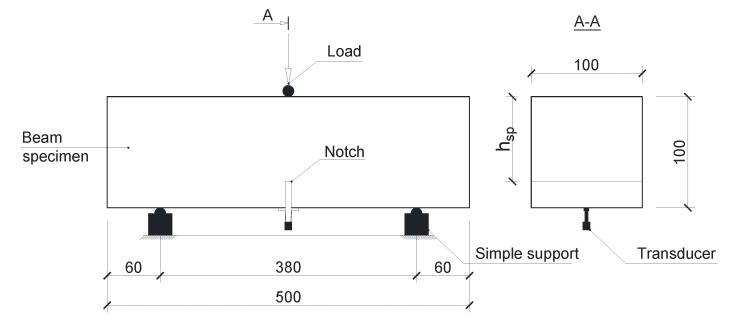
Dimensions and static scheme of the flexural strength test (unit: mm).

**Figure 2:**
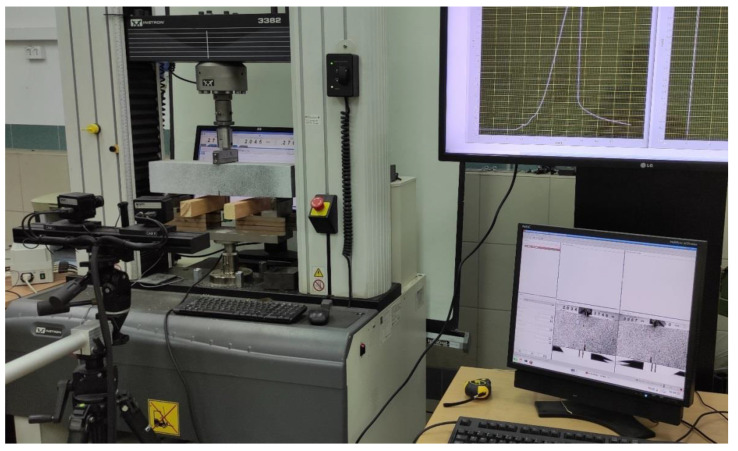
View of the test stand with the use of the ARAMIS 2M system by GOM.

From each of the five concrete compositions, three beams with a length of 500 mm and a square cross-section of 100 mm × 100 mm were also made. Notches not exceeding 4 mm wide and 15 mm deep were cut in the middle of the span in each of the beams. These samples were loaded in a three-point bending test according to the scheme recommended in PN-EN 14651: 2007. Each sample was placed in an Instron 3382 class 0.5 machine with a range of 100 kN that meets the requirements of the EN 12390-4 standard. The static scheme of the test is shown in Figure 1, and an exemplary sample placed in the machine, along with the entire DIC measurement system discussed, is shown in Figure 2.

Due to the aim of the study, i.e., the observation of the development of cracks on the side surface of the sample, the loading process was controlled by the constant speed of the head displacement while loading the sample. During the test, the displacement of the head loading the sample (in mm), the response of the sample to the given displacement in the form of reaction force (in kN) and crack mouth opening displacement (*CMOD*) (in mm) in the middle of the sample span were recorded. *CMOD* was recorded using a sensor for measuring the increase in crack opening with a range of 10 mm with a resolution of 0.01 mm. Each sample was loaded with a head moving speed of 1 mm/min. This method of loading corresponds to the *CMOD* growth rate not exceeding 0.05 mm/min, i.e., recommended in the PN-EN 14651: 2007 standard. Readings were recorded at a frequency of 1 Hz.

### 3.2. Digital Image Correlation Application

Complementing the research described in the previous subsection, the crack development process was monitored on selected samples using the optical image correlation system (DIC). The research used the ARAMIS 2M [36] optical image correlation system produced by the German company GOM. The system consists of two cameras with a focal length of 17 mm and a photo speed of 12 Hz with a resolution of 1600 × 1200 pixels, i.e., 1.92 Mpix. This system is designed for non-contact measurements of displacements and strains in flat or slightly curved elements subjected to static loads. The system prepared for operation is shown in Figure 2. With such a camera arrangement, the observation area of the displacement field on the sample was around 180 mm × 100 mm. Therefore, a 150 mm × 110 mm standard plate was used for calibration of the system. With this reference plate, the resolution of the readout in the camera image is equal to 0.04 pixels, and as a consequence the measurement resolution of the position of any point in the analyzed area is about 4 μm.

The process of tracing the development of cracks in the tested elements using the ARAMIS system [36] was carried out in two stages. In the first stage, during the three-point bending test of a given sample, two images were recorded every 1 s. These images were taken so that each pair of them could be assigned values of force acting on the sample, testing machine head displacement and *CMOD*. In the second stage, after completion of the research, thanks to the computational properties of ARAMIS software [36], changes in the observed displacement field were calculated. As a result of these measurements, at selected time moments, the previously obtained sets of results: time, displacement, force, *CMOD* were supplemented with the sum of the lengths of the resulting cracks.

It is worth underlying that the test of three-point bending of the prismatic sample with a notch, in which the LOP and mean residual flexural tensile stresses in *CMOD* function are determined is perfectly suited for its supplementation by measuring displacements on the side surface of the sample using DIC and subsequent interpretation of the obtained results in the light of the validation needs for constitutive models of the fracture mechanics. The analysis of the force–displacement or force–*CMOD* relationship does not allow for a comprehensive assessment of a very complex process, which is cracking of concrete reinforced with fiber, and that is why the DIC system is needed.

### 3.3. Cracks Determination and Measurement Technique

The measurement of the crack length at selected moments was made by analyzing the images taken by the ARAMIS system cameras with the superimposed calculation results, i.e., the interpretation of the test results in the form of contour graphs. The image of horizontal displacements is best suited to accurately identify the course of a single crack, but unfortunately it is not possible to determine its length on it, because at its apex the difference in displacements between points on both sides becomes too small. Determination of the length and course of many cracks is possible only with the analysis of strains—maximum principal or equivalent strains. Although the course of the crack in the strain graph is not shown as precisely as it happens in favorable conditions in the displacement graph, it is possible to analyze many cracks of any course and easily determine the location of their ends. In this paper, it was decided to use the graphs of the Mises equivalent strains [39], calculated according to the formula ε*_M_* = [(2/3)(ε_1_^2^ + ε_2_^2^ + ε_3_^2^)]^0.5^, where ε_1_, ε_2_ and ε_3_ represent the logarithmic principal strains. For the purposes of analyzed measurements, it was decided to divide the visible cracks into macro-cracks and micro-cracks.

**Figure 3:**
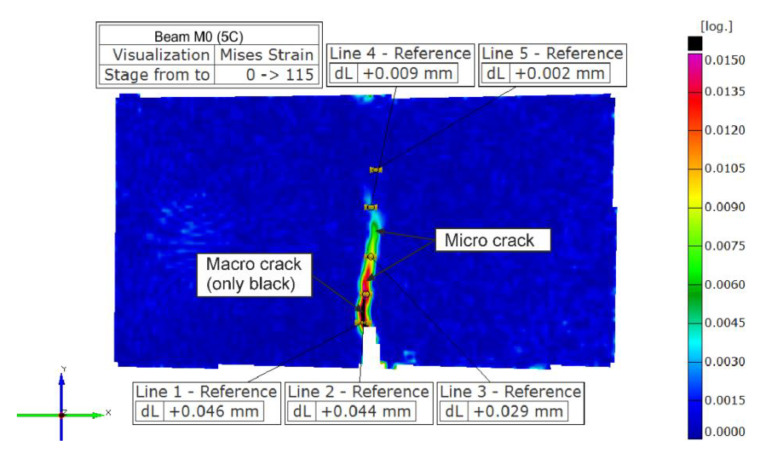
Interpretation of what we consider a macro-crack—on the example of material M0.

Figure 3 shows an exemplary contour graph of the equivalent Mises strains, which explains the adopted notations. In the discussed figure, the regions in which the value of these strains was at least 0.015 (black sub-region) were interpreted as macro-cracks. The remaining areas, also distinguishing from the background (contours from light blue to pink), were considered as micro-cracks. Only macro-cracks were measured to characterize the crack net development. The value of 0.015 was determined on the basis of the customary crack width of 50 μm, i.e., the allowable width of the crack in tight reinforced concrete structures. As shown in Figure 3, such a crack width corresponds to the equivalent von Mises strains of the value of just about 0.015.

The exact location of the crack inside the area with appropriate strains was determined on the basis of the crack course visible in the images, and if it was not possible, as the center line of the black region. In the case where the crack opening was very large (image registered at the end of the test), the measuring line was run along the selected edge of the crack. The subsequent images were interpreted jointly, i.e., if one of them showed the position of a part of the crack with certainty, the position was also copied onto the other images, assuming that although the length or opening of the crack may change, its position should remain unchanged. The method of measurement used is, of course, largely dependent on the researcher’s interpretation, but it is sufficiently accurate to determine when the changes in length were the greatest or monotonic, and in which samples the length of the crack net was the highest.

## 4. Experimental Results and Discussion

### 4.1. Analysis of Standard Tests

**Figure 4:**
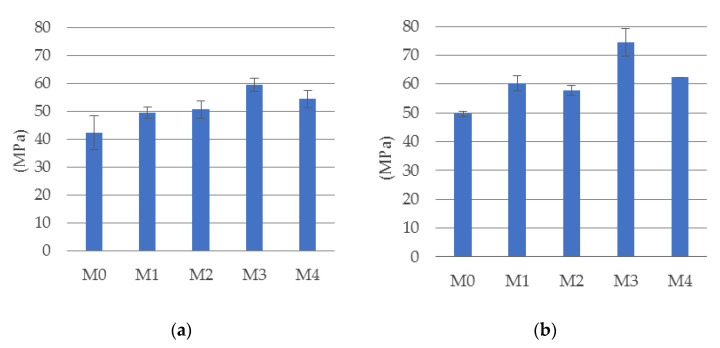
The mean compressive strength of cubic samples at (**a**) 28 and (**b**) 56 days.

**Figure 5:**
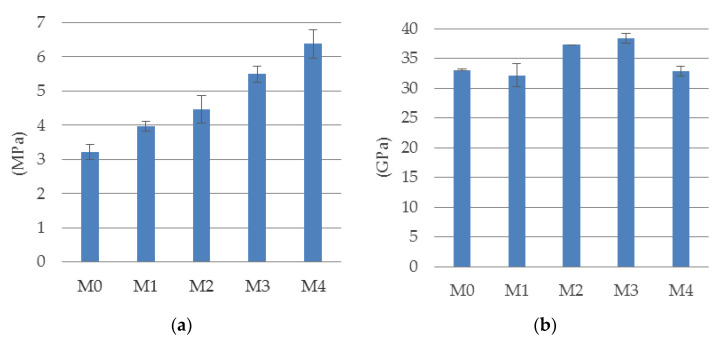
(**a**) Splitting tensile test results (on cubic samples); (**b**) stiffness modulus, both after 28 days.

The results of the standard tests discussed at the beginning of Section 3.1 are shown as graphs in Figure 4 and Figure 5. Figure 4 shows the results of the compressive strength tests after 28 and 56 days of the materials tested. Figure 5a shows the results of the tensile splitting test, and Figure 5b shows the values of the modulus of elasticity determined on the cylindrical specimens.

The analysis of the results of compressive tests (Figure 4) allows to state the normal increase in the strength of all materials with the maturation of the concrete. Each result was obtained as the average value from four tests, and the bars indicate standard deviation determined with 68% level of confidence (this remark apply to all experimental results presented in that paper). Out of the five materials tested, the material M3 showed the highest compressive strength, while the comparative material M0 showed the lowest. The relative difference between the strengths of these materials was almost 40%. The increase in the compressive strength of concrete with the percentage increase in its fiber content is related to the characteristic failure mode developing at the final stage of sample compression test. With a high content of steel fibers (M4 composition), the compressive strength was slightly worse than in the composition with a smaller amount of fibers (M3).

This is a typical effect observed in connection with the problem of effective homogenization of concrete at high fiber dosages, which may lead to local disturbances in the structure of the samples. Many researchers report about the different effects of fibers on compressive strength of composite materials. Larsen and Thorstensen [39] show that at a point, increasing the fiber content could have an adverse effect on the compressive strength. Meng and Khayat [40] reported this effect when the fiber content exceeded 3% by volume. This negative effect was explained by fiber agglomeration leading to entrapped air (Le Hoang and Fehling) [41]. Babar Ali et al. [42] notice that the positive effect of steel fibers on compressive strength is observed only at small doses and at a high-volume dose of fibers the effect is negative. Chajec and Sadowski [31] proved that the polypropylene fibers addition leads to nonsignificant changes in the value of compressive strength of concrete, whereas the addition of steel fibers to the concrete mix leads to a decrease in compressive strength when compared to the concrete without reinforcement.

By analyzing the results of the tensile splitting tests (Figure 5a) it can be concluded that the tensile strength increases with the amount of added fibers. The maximum addition of fibers (within the range considered in this paper) causes an almost twofold increase in tensile strength. Moreover, analyzing the results of the research on the modulus of longitudinal elasticity (Figure 5b), it was found that the highest value of the modulus was obtained for the M3 material.

The fact that the modulus of stiffness in the material with the highest amount of reinforcement fibers (M4) is at the same level as in the case of concrete without reinforcement, i.e., material M0, confirms that the difficulty in homogenizing a mixture with a high fiber content has a negative impact on the mechanical properties of the concrete. It is worth emphasizing that in the case of the tensile strength and modulus of elasticity, the uncertainty error of their determination at the 95% confidence interval is significantly lower than in the case of the compressive strength. The results of the experiment are in accordance with many published results. All researchers confirm a significant increase in tensile strength as a result of adding fibers to the concrete but Ding [43] pointed-out that the strengthening effect of steel fiber on axial tensile strength is greater than that of splitting tensile strength. Suksawang et al. [44] reported that fibers generally do not affect the modulus of elasticity but there are circumstances when the elastic modulus decreased with an average reduction of down to 20%. This could be attributed to extra (open or closed) porosity brought on by the high addition of fibers.

**Figure 6:**
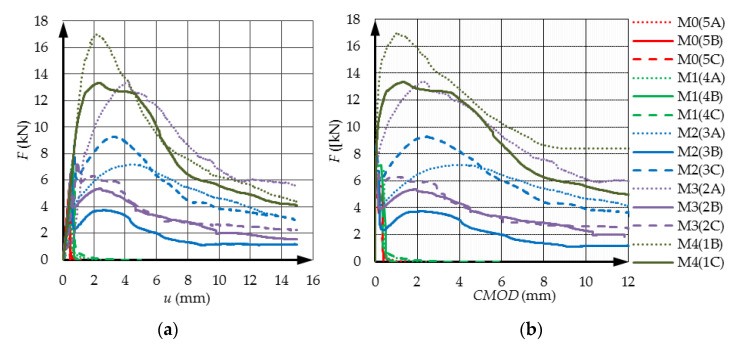
Equilibrium paths in the case of the tested materials and subsequent repetitions—force as a function of: (**a**) displacement; (**b**) crack mouth opening displacement (*CMOD*).

The results of the three-point bending tests performed as described in Section 3.1 are presented in the form of standard relationships: force–displacement (*F*-*u*)—Figure 6a and force–opening of notch (*F*-*CMOD*)—Figure 6b. These results were supplemented with the results of calculations of energy dissipated during the entire recorded bending process. Moreover, in Section 4.2 these results were supplemented with the results of cracks development measurements. The tensile strength in such test is determined on the basis of maximum force and corresponding displacement or *CMOD* value. The three-point bending test of prismatic samples is not only used to determine the so-called tensile strength in bending, but also to track the behavior of the material in the post-failure state, i.e., the equilibrium path from the initiation of the crack in the notch until the force drop to a value close to zero. Here, the value of *u* = 15 mm, and in the case of the *F*-*CMOD* relation, the value of *CMOD* = 12 mm was assumed. These limitations regarding the *CMOD* and deflection values result from the measuring range of the sensors used. The course and nature of discussed equilibrium paths very significantly depend on the type and amount of fibers used in concrete.

**Figure 7:**
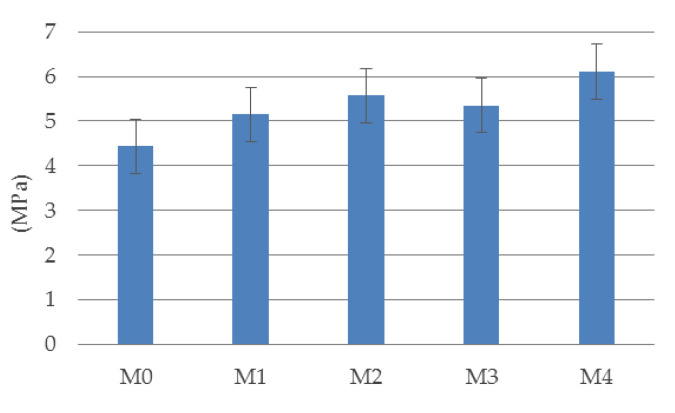
Limit of proportionality (LOP) of tested materials.

Based on the charts given in Figure 6, the LOP values for individual materials were determined in accordance with EN 14651. The results obtained in the form of a column chart are presented in Figure 7. By analyzing the chart, it can be concluded that the LOP stress values increase with an increase in the volume fraction of the dispersed reinforcement. In turn, Table 3 presents numerical values of residual flexural tensile strength calculated basing on graphs presented in Figure 6. The obtained results for the determined *CMOD* values determine the nature of the decrease in bending strength after crossing the extreme point in the *F*-*u* or *F*-*CMOD* graphs. Table 3 shows the maximum strength values (max (*Frj*)) obtained for the adopted *CMOD* values (bold characters). *Frj* is residual flexural tensile strength given by the expression $Frj=3Fj\cdot L/\left(2b\cdot h_{sp}{}^{2}\right)$, in which Fj is the load (force) corresponding with CMODj, L is the span length, b is the width of specimen, and $h_{sp}$ is the distance between tip of notch and the top of the specimen. It is worth noting that, for example, for the material M4, the size *Frj* (1.5 mm) = 11.5 MPa, which is almost twice as much as the determined LOP value for this material. For M0 and M1 materials, the bending strength after crack initiation for *CMOD* < 0.5 mm drops sharply to zero.

**Figure 8:**
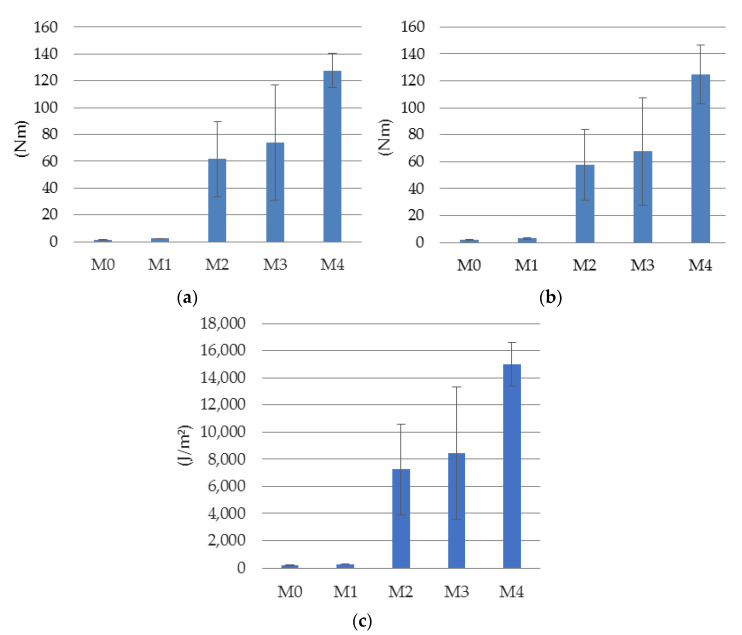
Energy dissipated during cracking—expressed the area under the curve: (**a**) *F*-*u* ($W_{0}^{u})$ and (**b**) *F*-*CMOD* ($W_{0}^{CMOD}$), as well as (**c**) fracture energy $G_{F}$.

The results shown in Figure 6 are the standard way of reporting the results of a three-point bending test on a notched specimen. These results can be supplemented by determining the size of the area under the *F*-*u* and *F*-*CMOD* graphs, which formally are the sum of the elastic energy (to crack) and the dissipated energy, i.e., energy dissipated during the development of macro- and micro-cracks in concrete. As the ratio of the elastic part is very small, the obtained values can be interpreted as a characteristic of the postcritical behavior of the fiber-reinforced material. These results are presented in Figure 8.

The dissipated energy $\left(W_{0}\right)$ in the beams during crack formation is calculated based on the area generated by the force-displacement curve *F*-*u* ($W_{0}^{u}$, *u* ranged from 0 to 15 mm, Figure 6a), or on the basis of the area limited by *F*-*COMD* curve ($W_{0}^{CMOD}$, *CMOD* ranged from 0 to 12 mm, Figure 6b). On the basis of $W_{0}^{u}$ also the fracture energy $G_{F}=\left(W_{0}^{u}+mg\delta \right)/\left(b\left(h-a_{0}\right)\right)$ [45] may be calculated, having in mind that m is the sample mass, g is gravitational acceleration, δ is deflection at final fracture (here assumed as 15 mm), b and h are the sample width and height, respectively, and finally $a_{0}$ is the depth of the notch. Of course, in that case also formula from JCI-S-001 [46], which is in accordance with RILEM procedures [47,48] and paper [49] may be also used. Nevertheless, in the case of M2 and M4 materials, both formulas are unjustified from the theoretical point of view and the obtained results should be treated as a kind of approximation. In the case of other materials, this approach leads to the determination of the fracture energy with a very high accuracy, as the fracture propagates almost along the axis of symmetry.

When analyzing the values of dissipated energy in fracture, the influence of steel fibers is clearly visible. Unreinforced concrete (M0) and concrete only reinforced with polypropylene fibers dissipate almost no energy after crack initiation, while in other cases these values are very significant. Additionally, there is a significant dispersion of the obtained results for the materials M2 and M3. The scatter of the results for the materials M0 and M1 is also significant; however, due to the significant differences in the values of $W_{0}^{u}$, $W_{0}^{CMOD}$ and $G_{F}$ for individual materials, it is not visible on the scale of the presented diagram. The dispersion in the case of the material with the highest degree of reinforcement is relatively small, which means that in the case of significant fiber content of the reinforcement, the postcritical behavior of the material is normalized.

Presented considerations are basically convergent with results reported in recent scientific literature. It was proved there that the maximum load and toughness are positively correlated with the fiber content and length, but have a negative correlation with the fiber diameter. What is more the addition of steel fiber makes the cracking mode of bending element gradually change from a single crack in the middle of the span to several approximately parallel cracks with an expansion to both ends of the segment [11]. The addition of steel fiber makes, the peak force, the deflection at failure, the fracture energy and the critical crack opening displacement increase [50,51].

### 4.2. Results of Three-Point Bending Tests with the Measurement of Crack Development Using DIC

The mean residual flexural tensile stresses in *CMOD* function or energy dissipated during cracking describe the cracking phenomena for the following materials determined in the three-point bending test will be supplemented with the analysis of the development of macro-cracks on the lateral plane of the samples using the optical image correlation system. It is worth emphasizing that, in general, cracks occur throughout the sample’s volume and their development is not always identical to that observed on the side surface. Nevertheless, the use of DIC allows for tracking only the development of the micro- and macro-crack system in a fairly large area and then to determine the total length of macro-cracks at each stage of the test, until it is completely destroyed.

**Figure 9:**
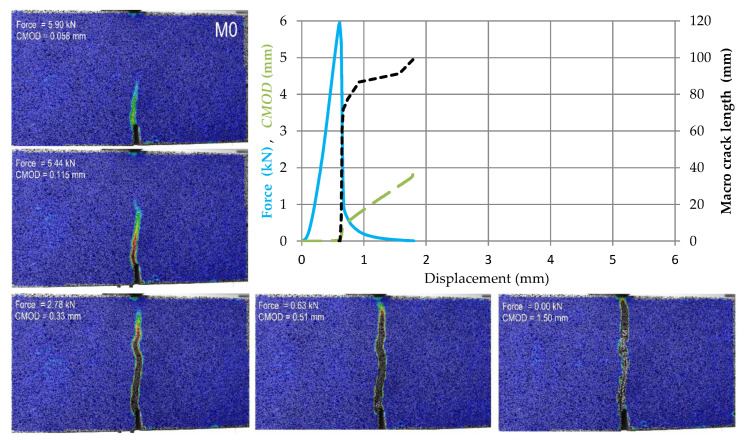
Force, *CMOD* and summary macro-crack length as a function of displacement in case of material M0 accompanied by cracks development in five chosen stages (contour plots of equivalent Huber–Mises strains).

**Figure 10:**
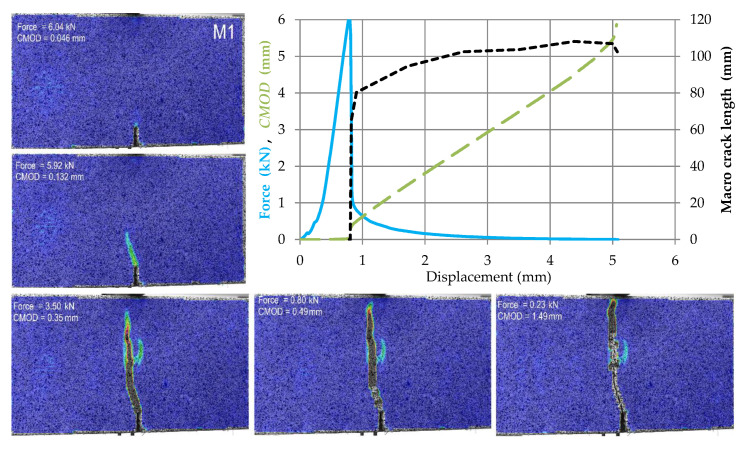
Force, *CMOD* and summary macro-crack length as a function of displacement in case of material M1 accompanied by cracks development in five chosen stages (contour plots of equivalent Huber–Mises strains).

**Figure 11:**
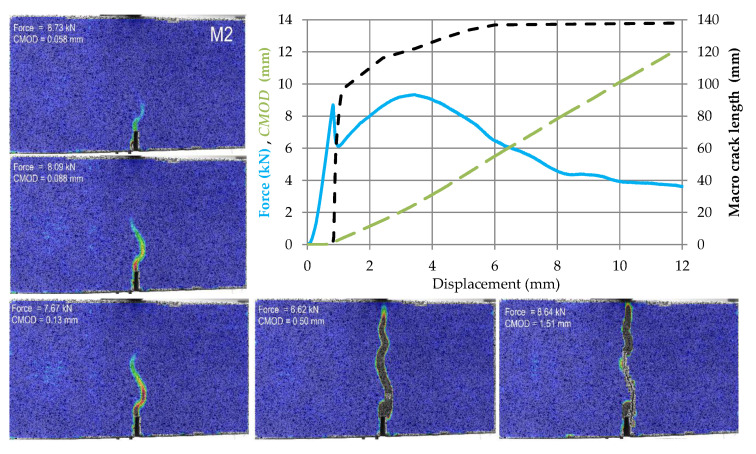
Force, *CMOD* and summary macrocrack length as a function of displacement in case of material M2 accompanied by cracks development in five chosen stages (contour plots of equivalent Huber–Mises strains).

**Figure 12:**
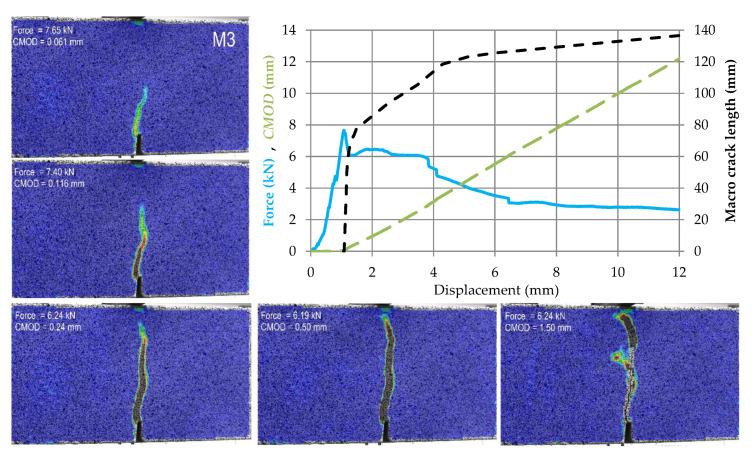
Force, *CMOD* and summary macrocrack length as a function of displacement in case of material M3 accompanied by cracks development in five chosen stages (contour plots of equivalent Huber–Mises strains).

**Figure 13:**
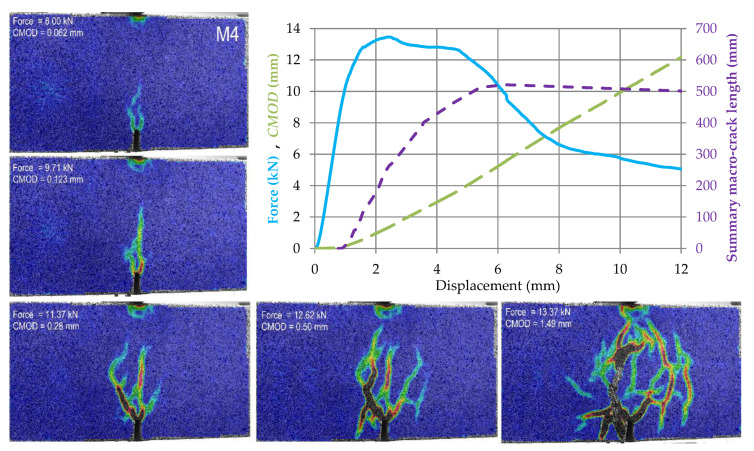
Force, *CMOD* and summary macrocrack length as a function of displacement in case of material M4 accompanied by cracks development in five chosen stages (contour plots of equivalent Huber–Mises strains).

The test results for subsequent samples made of M0–M4 materials are shown in Figure 9, Figure 10, Figure 11, Figure 12 and Figure 13. Each of these graphs shows a plot of force, *CMOD* and crack length versus the displacement of the loading head at the middle of the span. In these figures, the same scales on the axes were assumed for materials M0 and M1 (Figure 9 and Figure 10), then for materials M2 and M3 (Figure 11 and Figure 12), and a different force and *CMOD* scale for material M4 (Figure 13).

In addition, each of these figures shows five equivalent Mises strain contour graphs. The first one corresponds to the extreme of the obtained force, the second one is taken after 1 s from the previous one, i.e., the displacement increment by about 0.02 mm from the previous one, the third one with a *CMOD* about twice the value obtained in the previous image, the fourth one corresponding to a *CMOD* of about 0.5 mm, and the fifth one corresponding to a *CMOD* of about 1.5 mm.

In the case of the M0 material, i.e., without the use of reinforcement, the development of the macro-crack is very rapid and already at a displacement close to 2 mm, we can observe the separation of the sample into two parts. The macro-scratch is formed at the point where the incision is made and the shortest path is towards the top of the section, see Figure 9.

In the case of material M1, the development of the macro crack is not as rapid as for material M0, but its total length is at a similar level. The beam is completely separated into two parts for a displacement of more than 5 mm, cf. Figure 10. It is also worth noting that after initiating a fracture with a force value of about 4 kN, its gradual development takes place up to a displacement of about 3 mm. The nature of the crack development observed in the contour graphs of the Huber-Mises equivalent strains is analogous to the previous case, however, the formation of the main macro-crack is accompanied by the formation of a micro-crack with different development directions.

In the case of M2 material, shortly after the crack initiation, the element is strengthened with a simultaneous increase in *CMOD* and crack length. After reaching the second extreme of the *F*-*u* function, we observe the stabilization of the crack length with a continuous increase in *CMOD*. At the same time, it can be observed that the element does not lose its load bearing capacity despite the *CMOD* increase. At the maximum displacement shown in the graph (Figure 11), the load carried by the element does not fall below 40% of the observed maximum force value.

In the case of M3 material, shortly after the crack initiation, the force transmitted by the element stabilizes at approximately 75% of the maximum test force obtained. Then, despite the uniform increase in *CMOD*, the macro-crack development process clearly slows down. In addition, micro-cracks are formed.

In the case of the M4 material, the development of the macro-crack is accompanied by the formation of a whole network of micro-cracks, cf. Figure 13. It can be concluded that only the introduction of such an amount of fibers into the material completely changes the nature of the sample’s work at failure in the three-point bending test. Analyzing the contour plots of the Huber–Mises equivalent strains in accordance with the adopted interpretation in relation to the cracks, one can see a clear redistribution of deformations as a function of increasing displacement. One can see how the micro-cracks formed at earlier stages of the load in selected sub-regions close to reveal themselves in other places. The total length of the macro-cracks reaches an extreme value for a displacement close to 6 mm and then it slightly decreases to the end of the displacement measuring range. The total length of the macro-crack in the M4 material is over 500 mm and is nearly 4 times longer than in the case of M2 and M3 materials. At the end of the displacement measuring range (12 mm), the force value still exceeds 4.5 kN and is approximately twice as high as for materials M2 and M3.

### 4.3. Discussion of Obtained Results

Summarizing the results, it can be noted that insignificant volume fraction of dispersed reinforcement fibers slightly changes the failure mechanism of sample in the three-point bending test of notched beams, compare the M0 materials with M1, M2 and M3. In the case of the analyzed materials, it was only the reinforcement with a volumetric fraction of 1.1 percent that caused the failure mechanism of the sample made of M4 material to be radically different from the others. In this case, there are not only macro-cracks, but also micro-cracks, which may open or close during the growth of displacements depending on the local redistribution of the strain state.

The values of dissipated energy in fracture are determined with a relatively large statistical dispersion in the case of materials without reinforcement and slightly reinforced. In the case of M4 material with the highest degree of reinforcement, the dispersion is much smaller. This means that the dispersed fiber reinforcement stabilizes the post-critical behavior of the material. In the case of material M4, cracks develop even before maximum strength is reached. Such a large amount of steel fibers may also have negative effects: impede the proper preparation of the mixture (air voids, uneven density of the mixture, distribution of steel fibers and aggregates of various fractions), and as a consequence (diversifying the way the material works and introducing non-homogenous stress distribution) facilitate the formation of cracks on the surface samples. As a result, above a certain fiber volume content, although the load-bearing capacity increases, the tightness decreases.

**Figure 14:**
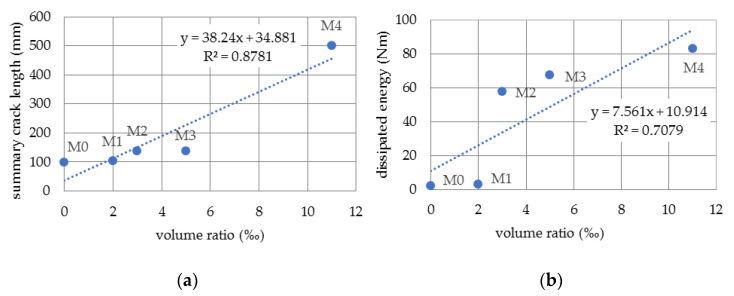
Correlation between (**a**) summary crack length, (**b**) dissipated energy and fiber volume ratio.

By analyzing the obtained results, an attempt was made to assess the correlation between the volume fraction of fibers in the analyzed materials and the total final macro-crack length and the energy dissipated in the fracture process. The obtained results are presented in the form of graphs in Figure 14.

The coefficient of correlation (R^2^) in the first case has a very significant value, i.e., it is equal to 0.88, while in the second case it is 0.71. It should be emphasized that the presented correlations were determined on a very limited set of the results of experimental tests and in a limited range of the total volume fractions of polypropylene and steel fibers.

## 5. Conclusions

The analyzed material compositions were proposed as material solutions for the construction of precast elements for subway tunnel linings. The introduction of dispersed reinforcement in the form of polypropylene fibers and steel fibers significantly affects all the mechanical properties of the resulting composite materials. The assessment of the behavior of material in a pre-critical and critical state is a research topic that has been developed for years with the use of various research and analytical techniques.

However, it is not easy to characterize the material after exceeding the critical state, mainly due to the rapidity of the material destruction process or the inability to use conventional measuring devices that could be damaged. For this purpose, non-contact measurement techniques of the entire displacement and strain fields based on the ARAMIS [39] digital image correlation system were used in the study. This system allowed for the observation of the development of micro- and macro-cracks in the entire load range. On the basis of presented experimental results, the following main conclusions can be stated as:

1. The use of digital image correlation methods allows not only qualitative but also quantitative assessment of the behavior of materials in post-critical states, especially in the case of the assessment of the development of cracks in the material.
2. Thanks to the use of DIC, it is possible to distinguish micro- and macro-cracks and to observe the redistribution of the strain state during the entire loading process.
3. The introduction of a new parameter, which is the total length of the macro-crack, allows for a quantitative evaluation of the course of the cracking process of concretes reinforced with dispersed fibers. Results of this nature can be used to calibrate the constitutive models of the fracture mechanics, e.g., by using FEM and inverse analysis.
4. The study showed a significant correlation between the total length of macro-cracks and the total volume fraction of reinforcement fibers in the material (R^2^ = 0.88). This conclusion was formulated on the basis of very limited data; therefore, its correctness should be verified in a wider research campaign.
5. A certain correlation was also observed between the energy dissipated during the fracture process and the total volume fraction of the fibers. The coefficient of determination in this case has a lower value (R^2^ = 0.71).

The approach to the crack resistance assessment of concrete reinforced with steel and polypropylene fibers presented in the paper can also be extended to concretes with other types of fibers (such as glass, carbon or vegetable fibers). It is worth noting that its useful-ness is enhanced in the case of concretes with a high degree of reinforcement, where the resulting crack pattern is irregular and complex. The authors plan further research on the topic under consideration, including the use of the tested concrete for a specific product in the form of reinforced concrete tubing for the lining of subway tunnels and tests on a semi-technical and technical scale. At the same time, we would also like to try to apply the obtained results to the validation of constitutive models of concretes available in commercial computational systems of the finite element method.

## Figures and Tables

**Table 1:** The properties of fibers used in the experiment.

Material	Shape	Length mm	Diameter µm	Tensile Strength N/mm^2^	Elasticity Modulus N/mm^2^	Density kg/m^3^
Polypropylene	Straight and bunch	12 ± 1.5	34 ± 1.7	365	4800	910
Steel fiber 4D-80/60BG	Hooked end	61 ± 2	750 ± 20	1800 ± 270	200,000	7850

**Table 2:** Mix compositions of all tested materials including the volumetric content of fibers.

Compound	Mix Composition kg/m^3^				
	M0	M1	M2	M3	M4
Cement	410	410	410	410	410
Sand 0/2 mm	650	650	650	650	650
Granite 2/8 mm	748	748	748	748	748
Granite 8/16 mm	410	410	410	410	410
Water	163	163	163	163	163
Superplasticizer	4.1	4.1	4.1	4.1	4.1
Polypropylene fibers	0	1.64	0	1.64	1.64
Steel fibers 4D-80/60BG	0	0	25	25	74
Volumetric content of fibers, (PP + S)%	0	0.2 + 0	0.3 + 0	0.2 + 0.3	0.2 + 0.9

**Table 3:** The mean residual flexural tensile strength corresponding with *CMOD*.

*CMOD* (mm)	M0	M1	M2	M3	M4
	*Frj* (MPa)	*Frj* (MPa)	*Frj* (MPa)	*Frj* (MPa)	*Frj* (MPa)
0.5	0.2	1.0	3.4	4.9	10.8
1.5	0.0	0.1	4.6	5.7	11.5
2.5	0.0	0.0	5.0	5.9	10.6
3.5	0.0	0.0	4.9	5.3	10.0
4.0	0.0	0.0	4.7	5.0	9.5

## Data Availability

Data sharing is not applicable to this article.
